# Circulating Dipeptidyl Peptidase Activity Is a Potential Biomarker for Inflammatory Bowel Disease

**DOI:** 10.14309/ctg.0000000000000452

**Published:** 2022-01-19

**Authors:** Simone E. Jaenisch, Catherine A. Abbott, Mark D. Gorrell, Peter Bampton, Ross N. Butler, Roger Yazbeck

**Affiliations:** 1 College of Medicine and Public Health, Flinders University, Bedford Park, South Australia, Australia;; 2 Flinders Health and Medical Research Institute, Bedford Park, South Australia, Australia;; 3 College of Science and Engineering, Flinders University, Bedford Park, South Australia, Australia;; 4 Liver Enzymes in Metabolism and Inflammation Program, Centenary Institute, Faculty of Medicine and Health, The University of Sydney, New South Wales, Australia;; 5 Department of Gastroenterology & Hepatology, Flinders Medical Centre, Bedford Park, South Australia, Australia.

## Abstract

**INTRODUCTION:**

Dipeptidyl peptidase (DPP)-4 is part of a larger family of proteases referred to as DPPs. DPP4 has been suggested as a possible biomarker for inflammatory bowel disease (IBD). Circulating DPP4 (cDPP4) enzyme activity was investigated as a potential biomarker for IBD. In addition, DPP enzyme activity and gene expression were quantified in colonic tissue of patients with IBD and non-IBD.

**METHODS:**

In study 1, DPP enzyme activity was quantified in plasma samples from 220 patients with IBD (Crohn's disease [CD] n = 130 and ulcerative colitis [UC] n = 90) and non-IBD controls (n = 26) using a colorimetric assay. In study 2, tissue and plasma samples were collected from 26 patients with IBD and 20 non-IBD controls. Plasma C-reactive protein (CRP) was quantified in all patients. Colonic DPP4, DPP8, DPP9, and fibroblast activation protein (FAP) gene expression was determined by quantitative polymerase chain reaction. cDPP and cFAP enzyme activity was also measured. Sensitivity and specificity were determined by receiver operating characteristic curve analysis.

**RESULTS:**

In study 1, total cDPP activity was found to differentiate patients with CD with active disease (n = 18) from those in remission (n = 19; sensitivity 78% and specificity 63%). In study 2, total cDPP and cFAP activity was 28% and 48% lower in patients with elevated CRP (>10 mg/L), respectively, compared with patients with normal CRP. Gene expression of DPP4, FAP, and DPP8 was also significantly higher in colonic biopsies from patients with IBD compared with non-IBD patients (*P* < 0.05).

**DISCUSSION:**

Our findings implicate the DPP enzyme family in intestinal inflammation and suggest future biomarker applications to differentiate the pathophysiological aspects of IBD.

## INTRODUCTION

Inflammatory bowel disease (IBD) is a chronic and debilitating condition characterized clinically by relapsing symptoms of diarrhea, rectal bleeding, abdominal pain, and fatigue (1,2). Several serological biomarkers are used to assist in the diagnosis and classification of IBD, monitoring of patient response to treatment, and disease severity (3). Colombel et al. (4) reported that frequent monitoring of plasma C-reactive protein (CRP) and fecal calprotectin, alongside the Crohn's Disease Activity Index (CDAI), was associated with higher rates of mucosal healing because of earlier and more intensive clinical management compared with using CDAI alone. A better understanding of the pathogenesis of IBD will help to uncover new biomarker candidates that may assist in improving disease management.

Dipeptidyl peptidase (DPP)-4 is a ubiquitously expressed serine protease and archetypal member of the larger DPP4 gene family of structurally homologous enzymes that we refer to here as DPPs and consists of DPP4, fibroblast activation protein (FAP), DPP8, and DPP9 (5,6). DPP4 and FAP are transmembrane proteases, whereas DPP8 and DPP9 are intracellular (7). In the liver and gastrointestinal tract, DPP4, DPP8, and DPP9 are localized predominantly to lymphocytes and epithelial cells (8,9). The DPP4 family shares similar DPP4-like enzyme activities, specifically cleaving a dipeptide from the N-terminus of a protein substrate that has a proline or alanine in the penultimate position (10). DPP4 cleaves a number of regulatory peptides, including the intestinotrophic growth factor glucagon-like peptide-2 (11), neuropeptides, and chemokines, and has been implicated in immunoregulation (5). Inhibition of DPP4 activity using selective and nonselective inhibitors has been associated with improvements to disease severity in mouse models of colitis (12–14).

DPP4 enzyme activity and expression have been investigated as a potential disease biomarker for a range of diseases, including IBD (15,16). Xiao et al. (17) reported decreased circulating DPP (cDPP) activity in patients with IBD compared with healthy patients. A more comprehensive study by Hildebrandt et al. (18) found that cDPP activity inversely correlates with disease activity scores and inflammatory markers in patients with CD and ulcerative colitis (UC). The chromogenic-derived and fluorometric-derived substrates, H-Ala-Pro- and H-Gly-Pro, have been the most commonly used synthetic substrates used to measure DPP enzyme activity. However, they are not selective for DPP4 and can also be hydrolyzed by the other DPPs—DPP8, DPP9, and FAP (19,20). Without the use of a selective DPP4 inhibitor, it is reasonable to deduce that these early studies were reporting total DPP enzyme activity and not DPP4 specific activity.

To further elucidate the potential of DPP4 enzyme activity as a biomarker for IBD, the aim of this study was to quantify DPP4 specific activity in bio-banked plasma samples from patients with CD and UC. A prospective observational cohort study was also undertaken to specifically quantify DPP enzyme activities and gene expression in the colonic tissue of patients with IBD and non-IBD patients and examine their relationships to disease activity.

## METHODS

### Study 1: cDPP activity in IBD

Plasma samples were obtained from the Gastroenterology and Hepatology Department at the Flinders Medical Centre, South Australia. Plasma samples had been collected from patients with CD and UC during routine clinic visits between 2008 and 2015 and were stored at −80°C. Clinical notes included physical examination, patient general well-being, and blood analysis. These samples were approved for research use by the Flinders Clinical Research Ethics Committee (Study ID 13/08).

Venous blood was also collected from healthy adult donors presenting for blood donation (for routine diagnostic services) at the Women's and Children's Hospital under the guidelines and approval of the Women's and Children's Health Network Research Ethics Committee. After collection, blood samples were centrifuged (800*g* for 30 minutes) to separate plasma from cellular components, and plasma was stored at −80°C.

### DPP enzyme activity measurement

DPP enzyme activity in human plasma samples was quantified by a kinetic colorimetric enzyme assay as previously described (13). The DPP substrate, H-Ala-Pro-pNA (extinction coefficient: 9,450 M^−1^ cm^−1^; Bachem, Switzerland), was used at a final concentration of 1 mM in 0.1 M sodium phosphate buffer for all enzyme assays. Dual absorbance readings were used to correct for any effect of hemolysis. Measurements were obtained at 405 and 600 nm every 10 minutes for a total of 90 minutes at 37°C using the CLARIOstar(BMG Labtech, Germany). All samples were analyzed in triplicate. Total DPP (tDPP) enzyme activity enzyme activity was defined as 1 μmol of pNA produced/min/L of plasma and expressed as U/L at 37°C.

To discriminate between DPP4 activity and that of other DPPs, the selective DPP4 inhibitor, sitagliptin phosphate monohydrate (BioVision), was used in enzyme assays. The IC50 value for sitagliptin in human plasma has been reported as approximately 15 nM (21,22). A final concentration of 1 µM sitagliptin was therefore used to minimize any off-target effects to other DPP enzymes. The amount of activity inhibited by sitagliptin was termed “DPP4 activity,” and the remaining activity, which may contain DPP4, FAP, DPP8, and DPP9 activity, was referred to as “residual activity.”

### Study 2: DPP expression and activity in IBD

Patients with CD and UC, and non-IBD patients were recruited from the endoscopy clinic at the Flinders Medical Centre, South Australia, between December 2015 and April 2017. Exclusion criteria included patients younger than 18 years, those undergoing treatment with DPP4 inhibitors, and pregnant women. Study participants were recruited by a research nurse, and consent obtained on the day of the endoscopy procedure. All participants were fasted and had undergone bowel preparation before their colonoscopy. This study was approved by the Southern Adelaide Clinical Human Research Ethics Committee (SAC HREC 135.15).

Disease activity was determined endoscopically by the treating gastroenterologist and confirmed by routine pathology. Disease activity was also assessed by clinical disease scoring indices, the Harvey-Bradshaw index, and the Mayo index. Active disease was defined as a Harvey-Bradshaw (CD) score of >4 (23) or a Mayo score (UC) of >2 (24).

Forty milliliter blood was collected into di-potassium ehylenediaminetetraacetic acid blood collection tubes before endoscopy. Blood samples were centrifuged (800*g*, 20 minutes, room temperature) to separate plasma, white and red blood cells. Plasma was then aliquoted and stored at −80°C until analysis.

Up to 10 biopsies were collected from each patient with IBD and non-IBD patient. Inflammation was identified by the presence of edema, friability, erythema, granularity, and increased vascularity. Up to 5 biopsies were collected from inflamed tissue regions (colon or rectum) with up to 5 matched biopsies collected from adjacent inactive tissue regions and random inactive tissue sites. Biopsies from endoscopically normal tissues were collected from the colon and the rectum of non-IBD patients. Samples were stored in cryovials containing RNA-later.

### DPP enzyme activity measurement

Tissue biopsies were homogenized in ice-cold 10 mM Tris-HCl buffer (pH 8). Membrane and soluble cell fractions were separated by ultracentrifugation 2 times (88,000*g* for 30 minutes, 4°C; Optima Max-TL; Beckman Coulter, Indianapolis, IN), and membrane fractions were resuspended in ice-cold 10 mM Tris-HCl. Membrane and soluble fractions were then transferred into clean Eppendorf tubes and kept on ice for immediate analysis by enzyme assay.

DPP enzyme activity in plasma, membrane and soluble tissue fractions was measured using the method described for study 1. One μM of the DPP4 inhibitor, sitagliptin phosphate monohydrate, and 10 μM of DPP8/9 inhibitor, 1G244, were used to differentiate DPP4 and DPP8/9 specific activity. Caco2 cell extracts were used as quality controls for each assay. Total DPP enzyme activity was defined as 1 μmol of pNA produced/min/L of plasma and expressed as U/L at 37°C.

#### Protein assay.

Protein concentration in tissue samples was quantified using a modified Bradford assay as per manufacturer's instructions (Bio-Rad Laboratories, Hercules, CA). Absorbance was measured at 595 nm on a CLARIOstar plate reader (BMG Labtech, Ortenberg, Germany).

### FAP enzyme activity

Plasma FAP enzyme activity was measured using the fluorogenic substrate 3144-aminomethylcoumarin (25). One hundred microliters of reactions consisted of 5 μL of 1 in 5 diluted plasma, phosphate buffered saline and 150 μM aminomethylcoumarin substrate. The microplate was read in a POLARstar plate reader (BMG Labtech) at excitation of 355 nm and emission of 450 nm every 5 minutes for 1 hour at 37°C. Reference samples (3 sera) were used to normalize for intra-assay variations as described by Uitte et al. (26). Data were expressed as units per liter of plasma_._

### C-reactive protein

CRP is an acute phase reaction protein that is elevated in a number of inflammatory conditions, including sepsis and IBD (27). Frozen plasma samples were thawed at room temperature and clarified by centrifugation (7,500*g* for 10 minutes) to remove any debris. CRP was quantified using a particle-enhanced immune turbidimetric assay, and samples were measured using the Cobas 8000 modular analyzer series (Roche, Basel, Switzerland). CRP concentration was expressed as milligrams per liter plasma.

### Colonic DPP gene expression by quantitative polymerase chain reaction

Excess RNA-later was removed, and biopsies were weighed and homogenized in 2 mL round bottom snap-lock Eppendorf tubes using the TissueLyser bead mill system (Qiagen, Hilden, Germany). RNA was extracted using the RNA easy kit (Qiagen) according to the manufacturer's instructions. RNA was quantified using the spectrophotometer, NanoDrop 2000 (Thermo Fisher Scientific, Waltham, MA). RNA quality was assessed by 1% agarose gel electrophoresis.

Complementary DNA (cDNA) was synthesized from 1 μg of RNA using the QuantiTect cDNA synthesis kit (Qiagen). The yield of cDNA at the end of the reaction was approximated by assuming that 1 µg of RNA will yield 1 μg of cDNA. Gene expression was quantified in approximately 10 ng of cDNA using the real-time polymerase chain reaction (PCR) cycler, Rotor-Gene Q (Qiagen). Ten µL reactions consisted of Kappa Universal SYBR Green Mastermix 1x (Sigma Aldrich), sterile reverse osmosis water, and 100 nM of primer (see Supplementary Table 1, Supplementary Digital Content 2, http://links.lww.com/CTG/A743). Quantitative PCR (qPCR) reaction cycling conditions were as follows: 1 cycle at 95°C for 2 minutes, followed by 40 cycles at 95°C for 1 second, 65°C for 20 seconds, and 95°C for 15 seconds. Each sample was analyzed in triplicate, and each run contained a positive control, negative template control, and nonreverse transcriptase control. Melt curve analysis and 2% agarose gel electrophoresis were performed on qPCR products to assess the identity of PCR products and ensure the absence of contamination.

External gene standards consisting of purified PCR product of known concentration were included in qPCR runs to determine the exact gene copy number in patient colorectal biopsies. A minimum of 4 external gene standards ranging from 2 × 10^7^ copies to 2 × 10^1^ copies were included in each qPCR run to estimate the gene copy number. Standards were made using previously described methods (28,29). All gene expression data are presented as copies of the gene of interest normalized to the copies of housekeeping genes, TATA and peptidylpropyl isomerase A (*Ppia*) and expressed as arbitrary units (AU) (30).

### Statistics

Data normality was determined by the analysis of frequency histograms and quantile-quantile plots. Nonparametric comparisons between multiple groups for qPCR and enzyme activity data were made by the Kruskal-Wallis test with a Mann-Whitney post hoc and Bonferroni correction. Sensitivity and specificity of DPP enzyme activity as an IBD biomarker were determined by receiver operating characteristic (ROC) curve analysis.

Comparisons between active and inactive (remission) disease groups were made by the Mann-Whitney *U* test. For all analyses, *P* < 0.05 was considered significant. All data are expressed as median (interquartile range). Statistical comparisons were made using SPSS Statistics version 23 (IBM, Armonk, NY).

## RESULTS

### Study 1: patient characteristics

We included 220 plasma samples from patients with IBD in this study. There were 130 patients identified as CD and 90 patients as UC. A total of 37 patients with CD and 19 patients with UC were able to be characterized according to disease activity (active or remission; Table 1).

**Table 1.** Baseline patient characteristics for study 1

	Crohn's disease	Ulcerative colitis
Total no. of patients (n)	130	90
Age, mean (range)	37 (18–33)	50 (19–108)
Disease activity (active vs remission)	18 vs 19	9 vs 10
Resection history (previous resection vs no resection)	10 vs 16	14 vs 37

Disease activity and resection history were not available for all samples analyzed in study 1.

### cDPP activity is reduced in patients with CD and UC

Total DPP activity was 18% lower in IBD (15.1 [12.5] U/L plasma, n = 220) compared with non-IBD controls (17.7 [14.5] U/L plasma, n = 26, *P* < 0.05). The interassay coefficient of variation was 4.62%. Residual and DPP4 specific activity was also significantly lower by 17% and 12%, respectively, in patients with IBD compared with non-IBD controls.

**Figure 1.**
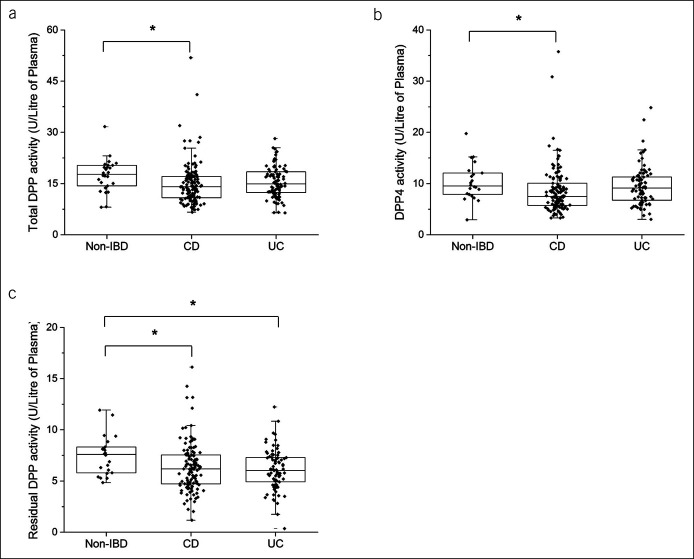
cDPP activity in non-IBD, CD, and UC patients. Enzyme activity was calculated as (**a**) total DPP (n = 26/non-IBD; n = 130/CD; and n = 90/UC), (**b**) specific DPP4, and (**c**) residual DPP activity. Each data point represents the enzyme activity of a single patient. Box-plot overlay represents the median and interquartile range, and whiskers represent the range excluding outliers. *P < 0.05 compared with non-IBD. CD, Crohn's disease; cDPP, circulating dipeptidyl peptidase; DPP, dipeptidyl peptidase; IBD, inflammatory bowel disease; UC, ulcerative colitis.

Total DPP activity was 20% lower in the plasma of patients with CD compared with non-IBD patients (*P* < 0.05; Figure 1a). Using sitagliptin, cDPP4 activity in plasma was found to be 21% lower in patients with CD compared with non-IBD patients (*P* < 0.05; Figure 1b). Residual DPP activity was also significantly lower by at least 18% in the plasma of patients with CD and UC compared with non-IBD patients (*P* < 0.05; Figure 1c).

### Patients with active CD and UC have differential cDPP activity profiles

**Figure 2.**
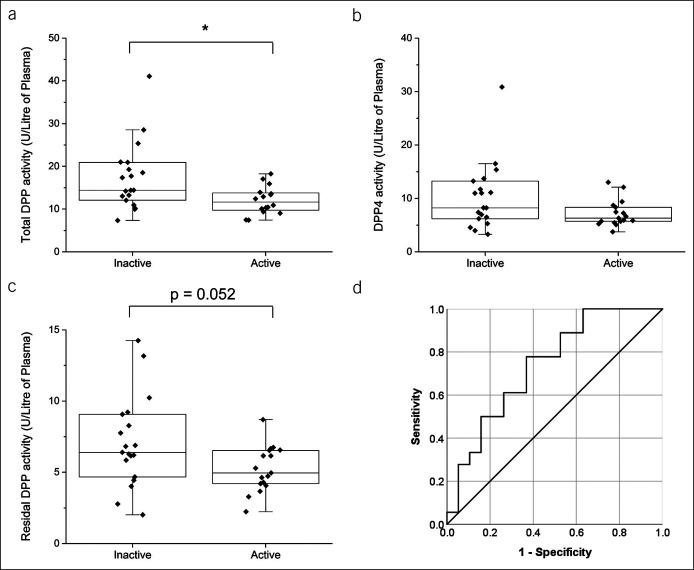
Reduced cDPP activity in active CD. cDPP activity in plasma was calculated as (**a**) total DPP, (**b**) specific DPP4, and (**c**) residual DPP activity (n = 16/active; n = 19/inactive). (**d**) Receiver operating characteristic curve analysis of sensitivity and specificity for total DPP4 activity to differentiate inactive and active disease. Each data point represents the enzyme of a single patient. Box-plot overlay represents the median and interquartile range, and whiskers represent the range excluding outliers. *P < 0.05 active vs inactive. CD, Crohn's disease; cDPP, circulating dipeptidyl peptidase; DPP, dipeptidyl peptidase; IBD, inflammatory bowel disease; UC, ulcerative colitis.

IBD patient cohorts were further classified into those with active and inactive disease at the time of blood collection. Total DPP activity was 30% lower in patients with CD with active disease compared with patients with inactive disease (*P* < 0.05; Figure 2a). However, DPP4 activity and residual DPP activity were unchanged between patients with CD with active and inactive disease (Figure 2b,c).

**Figure 3.**
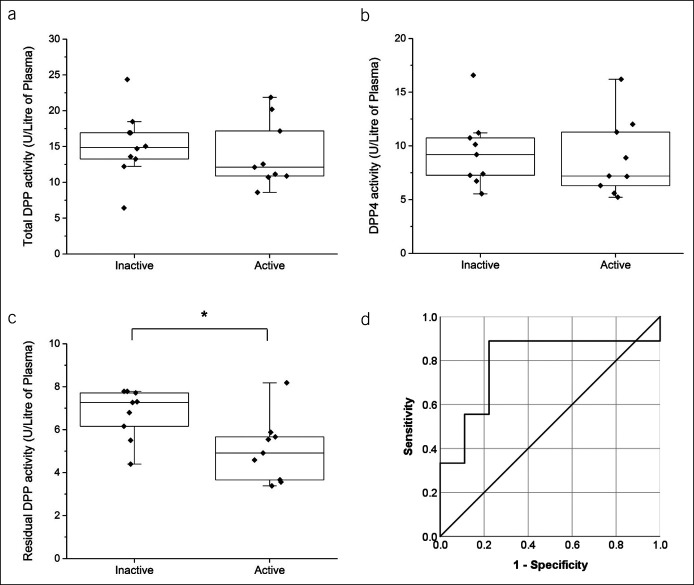
cDPP activity in patients with active and inactive UC. cDPP activity in plasma was calculated as (**a**) total DPP activity, (**b**) specific DPP4 activity, and (**c**) residual DPP activity (n = 9/active and n = 9/inactive). (**d**) Receiver operating characteristic curve analysis of sensitivity and specificity for residual DPP4 activity to differentiate inactive and active disease. Each data point represents the enzyme activity of a single patient. Box-plot overlay represents the median and interquartile range, and whiskers represent the range excluding outliers. *P < 0.05 active vs inactive. cDPP, circulating dipeptidyl peptidase; DPP, dipeptidyl peptidase; UC, ulcerative colitis.

There was no difference in total DPP activity (Figure 3a) and DPP4 activity (Figure 3b) between patients with UC with active disease and those in remission. However, residual DPP activity was significantly lower by 33% in patients with UC with active disease compared with those in remission (*P* < 0.05; Figure 3c).

Using the ROC curve analysis, it was found that a cutoff value of 14.1 U/L plasma total DPP activity differentiated patients with CD with active disease (n = 18) and in remission (n = 19), with a sensitivity of 78% and a specificity of 63% (area under the ROC curve [AUROC] = 0.74, 95% confidence interval 0.58–0.90, *P* = 0.01; Figure 2d). The positive and negative likelihood ratios for CD were calculated as 2.11 and 0.35, respectively.

In addition, a cutoff value of 6.02 U/L plasma residual DPP activity differentiated patients with UC with active (n = 9) and inactive (n = 9) disease, with a sensitivity of 89% and a specificity of 78% (AUROC = 0.79, 95% confidence interval 0.56–1.00, *P* = 0.04; Figure 3d). The positive and negative likelihood ratios for UC were 4.00 and 0.14, respectively.

### Study 2: patient characteristics

To build on the findings of study 1, a total of 46 patients (20 non-IBD, 13 CD, and 13 UC) were recruited into this prospective study (Table 2). Active disease was identified in 50% (5/10) of the patients with CD and 53.8% (7/13) of the patients with UC (Table 2).

**Table 2.** Baseline patient characteristics for study 2

	Non-IBD	CD	UC
Sample size (n)	20	13	13
Age, mean (range)	59 (30–77)	43 (25–79)	47 (27–69)
Sex (female/male)	6/14	7/6	5/8
Time since diagnosis, yr, mean (range)	—	15 (1–30)	11 (1–24)
Inflammation present during scope (%)	0	50	53.8
Disease activity indices			
Harvey-Bradshaw >4	—	3/13	—
Mayo score >2	—	—	5/11
IBD medications			
Immunosuppressants (azathioprine, methotrexate, and mercaptopurine)	1	8	3
Anti-inflammatory (mesalazine and sulfasalazine)	—	4	5
Biologicals (humira/infliximab and adalimumab)	—	2	3
Steroids	1	2	—
Combination therapy	—	6	2
Comorbidities and smoking status			
Diverticula/itis	11	—	3
Arthritis (osteoarthritis/psoriatic arthritis)	3	—	1
Type 2 diabetes	3	—	—
Previous cancer	3	2	—
Smoking status (current, ex-smoker, or never)	3, 8, 9	4, 2, 7	1, 3, 9

CD, Crohn's disease; IBD, inflammatory bowel disease; UC, ulcerative colitis.

### cDPP enzyme activity

Total cDPP activity and DPP4 activity did not differ between non-IBD, CD, and UC patients (Table 3). cDPP activity was also unchanged in IBD during active disease or by clinical disease activity indices (Table 3). Total plasma, specific DPP4, and residual activity did not differ by patient age, sex, or smoking status.

**Table 3.** Plasma DPP activity in non-IBD patients and patients with IBD (study 2)

	n	Total DPP activity (U/L)	DPP4 activity (U/L)	Residual activity (U/L)
Non-IBD	20	14.0 (12.7–17.2)	7.6 (6.3–10.3)	7.0 (5.7–7.8)
IBD (UC + CD)	23	13.0 (8.1–22.3)	6.3 (3.9–11.1)	6.9 (3.9–12.2)
CD	10	13.4 (11.0–16.6)	6.7 (4.6–8.5)	6.8 (6.1–8.1)
UC	13	14.4 (12.8–16.0)	6.5 (5.8–8.3)	7.2 (6.4–8.6)

DPP4-specific activity calculated using the DPP4 selective inhibitor, sitagliptin. Data are expressed as median (range).

CD, Crohn's disease; DPP4, dipeptidyl peptidase-4; IBD, inflammatory bowel disease; UC, ulcerative colitis.

### cDPP and cFAP activity is reduced in patients with elevated CRP

**Figure 4.**
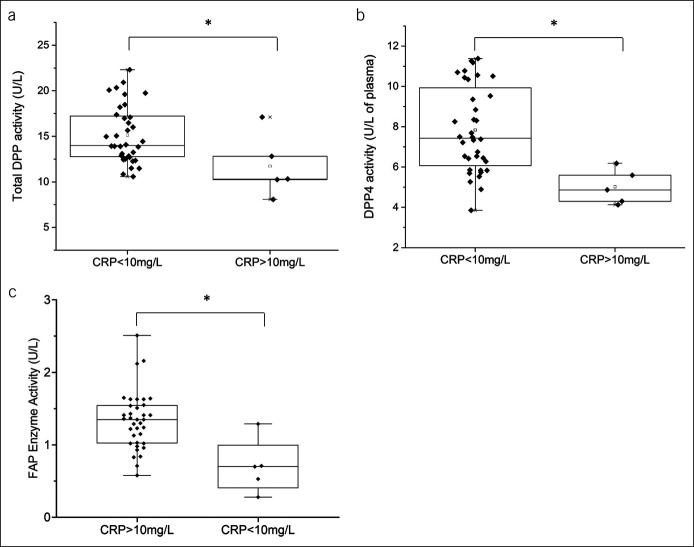
cDPP and FAP activity and CRP were compared in all patients. Plasma (**a**) total DPP, (**b**) DPP4, and (**c**) FAP enzyme activity were classified according to CRP <10 mg/L (n = 37; n = 18/non-IBD, n = 12/UC, and n = 7/CD) and CRP >10 mg/L (n = 5; n = 1/non-IBD, n = 1/UC, and n = 3/CD). Box-plot overlays represent median and interquartile range, and whiskers represent range. *P < 0.05, CRP <10 mg/L vs CRP >10 mg/L. CD, Crohn's disease; cDPP, circulating dipeptidyl peptidase; CRP, C-reactive protein; DPP, dipeptidyl peptidase; FAP, fibroblast activation protein; IBD, inflammatory bowel disease; UC, ulcerative colitis.

CRP is an acute phase reaction protein and was quantified as a subjective measure of inflammation in individual patients. Elevated plasma CRP (>10 mg/L) was found in 5 of 42 patients, which included 4 patients with IBD and a single non-IBD patient with diverticulitis. Total DPP activity was 28% lower in patients with elevated CRP (>10 mg/L) compared with patients with normal CRP (*P* < 0.05; Figure 4a). cDPP4 activity was also reduced by up to 34% in patients with elevated CRP compared with patients with normal CRP (*P* < 0.05; Figure 4b).

cFAP activity has also been identified as a potential biomarker of inflammatory and fibrotic conditions (26,31). In this study, cFAP activity was unchanged in plasma between non-IBD, CD, and UC patients. There was no difference in FAP activity between patients with IBD with active and inactive disease as determined by endoscopy. However, FAP activity was approximately 48% lower in patients with elevated CRP (>10 mg/L) compared with patients with normal CRP (*P* < 0.05; Figure 4c). Plasma FAP enzyme activity was also significantly correlated with cDPP4 enzyme activity (Pearson *r* = 0.663, *P* < 0.0001).

The ratios of plasma CRP:cFAP and CRP:cDPP4 were also compared. In patients with elevated CRP (>10 mg/L), CRP:cFAP was significantly higher (42.6 [22.5–139.6]) compared with patients with normal CRP (1.1 [0.3–2.8], *P* < 0.0005). Similarly, the ratio of CRP:cDPP4 was significantly higher (5.2 [3.5–11.8]) in patients with elevated CRP (>10 mg/L) compared with those with normal CRP (0.2 [0.1–0.5], *P* < 0.0005).

### Colonic expression of DPP4, FAP, and DPP8 mRNA is higher in IBD tissue biopsies

**Figure 5.**
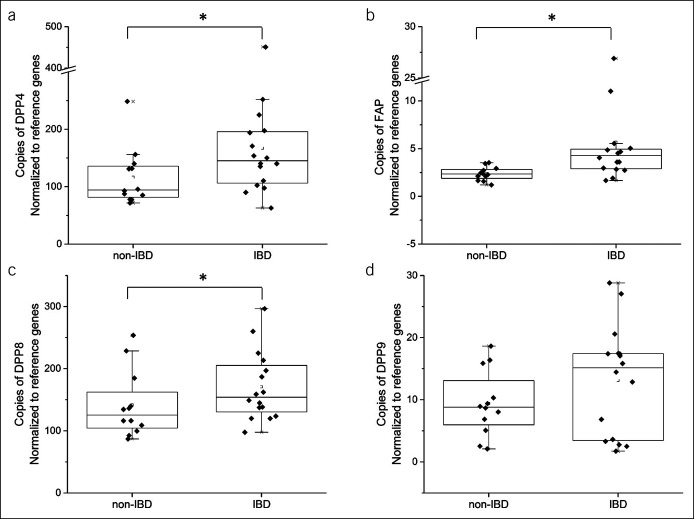
DPP mRNA expression in noninflamed colorectal tissue from non-IBD patients (n = 12) and patients with IBD (n = 16; n = 9/CD, n = 7/UC). Gene copy number for (**a**) DPP4, (**b**) FAP, (**c**) DPP8, and (**d**) DPP9 was determined by quantitative polymerase chain reaction. Gene copy number was expressed normalized to reference genes, *Ppia* and TATA. Box plots represent median and interquartile range, and whiskers represent range excluding outliers. *P < 0.05, non-IBD vs IBD. CD, Crohn's disease; DPP, dipeptidyl peptidase; FAP, fibroblast activation protein; IBD, inflammatory bowel disease; UC, ulcerative colitis.

DPP4 gene expression was 150% higher in normal colorectal tissue from patients with IBD compared with non-IBD patients (*P* < 0.05; Figure 5a). Higher expression of FAP (4.3 [2.9–5.2] AU vs 2.4 [2.0–2.8] AU, *P* < 0.05; Figure 5b) and DPP8 (154.0 [134.0–201.1] AU vs 125.4 [106.6–140.1] AU, *P* < 0.05; Figure 5c) was also found in colorectal tissue from patients with IBD compared with non-IBD patients, respectively. There was a strong correlation between DPP8 and DPP9 gene expression (Pearson *r* = 0.765, *P* < 0.0001).

### DPP enzyme activity in colorectal tissue

DPP8/9 activity was 2.2-fold higher in noninflamed colorectal tissue from patients with IBD compared with non-IBD patients (3.5 [2.3–4.3] U/mg of protein vs 1.1 [1.6–3.2] U/mg of protein, *P* < 0.05; see Supplementary Figure 1, Supplementary Digital Content 1, http://links.lww.com/CTG/A742). Total soluble activity, membrane activity, and specific tDPP4 activity in normal colorectal tissue were unchanged between non-IBD patients and those with IBD. However, when matched IBD patient samples of inflamed and noninflamed tissue were compared, total soluble tDPP activity was significantly higher in biopsies from active regions compared with inactive tissue (see Supplementary Figure 2, Supplementary Digital Content 1, http://links.lww.com/CTG/A742).

## DISCUSSION

To the best of our knowledge, this is the first study to measure DPP4, DPP8/9, and FAP specific enzyme activity in human IBD. Our findings suggest that total cDPP activity is reduced in patients with active CD. The AUROC curve suggests that total cDPP activity could have utility to differentiate active vs inactive disease in CD; however, additional validation studies are required to support this observation. Low cDPP and cFAP enzyme activity was associated with elevated plasma CRP, lending additional support for their use as serological markers of inflammation. In addition, we also observed higher gene expression of DPP4, FAP, and DPP8 in colorectal tissue of patients with IBD compared with non-IBD patients.

Enzymatically active, cDPP4 is believed to result from the enzymatic shedding of membrane DPP4 from multiple cell types, including adipocytes, smooth muscle cells (32), and cytotoxic T-lymphocytes (33–35). Casrouge et al. (34) previously described *in vitro*, in mouse models, and in human plasma samples that most of cDPP4 is derived from lymphocytes. Lettau et al. (35) reported that most enzymatically active DPP4 in circulation can be traced to the degranulation of cytotoxic T-lymphocytes. The decreased cDPP enzyme activity in active IBD observed in this study, and others could reflect the dysregulated IBD immune profile and aberrant activation of circulating lymphocytes (36). Alternatively, it has previously been suggested that decreased DPP4 activity in IBD could be a compensatory mechanism to prolong the half-life of the intestinotrophic growth factor, glucagon-like peptide-2 (17). Additional studies are needed to understand the pathophysiological mechanisms associated between circulating DPPs and active disease in IBD.

By contrast, less is known about the cellular origin of cFAP. Keane et al. (25) previously suggested that specific cell types may produce FAP for release into circulation, or FAP could be shed from the cell surface. Although we did not observe any significant difference in cFAP enzyme activity between patients with IBD and non-IBD patients, there was a moderate-to-strong relationship between cDPP4 and cFAP enzyme activity, suggesting an overlap in regulatory mechanisms of DPP4 and FAP expression and/or of release as circulating forms. Coexpression of FAP and DPP4 has been previously reported in some cell types, such as fibroblasts (37–39), and it is possible that these enzymes are being shed into circulation.

Previous studies in IBD (18,40) and rheumatoid arthritis (31,41) reported inverse correlations between cDPP and cFAP activity with inflammation, indicated by CRP concentrations. Consistent with our findings, Hildebrandt et al. (18) previously reported a relationship between cDPP activity in patients with IBD and CRP, orosomucoid as well as clinical indices, such as CDAI. Despite the limited number of study participants in our study with raised CRP, we found that both lower cDPP and lower cFAP were associated with elevated CRP (CRP >10 mg/mL). Interestingly, when the ratio of CRP to cFAP and cDPP4 were calculated, there was greater resolution between those with elevated CRP and normal CRP. CRP is an acute phase reaction protein that is released primarily from hepatocytes in response to increased concentrations of interleukin (IL)-6 and is elevated during tissue damage, infection, and inflammation (27,42). Although CRP can provide useful clinical information, CRP is only weakly associated with endoscopically detected inflammation in IBD (43,44). Larger, longitudinal studies are required to better define the relationship between lower cDPP and disease flare.

cDPP enzyme activity has been previously investigated as a serology marker in several paradigms, including chronic inflammatory diseases (26,31), cancer (45,46), and psychosocial disorders (15,47). cDPP4 is heavily glycosylated, shielding it from proteolytic attack (48). Because of the stability of cDPP4, it could have utility as an acute phase biomarker of active disease in patients with IBD. Hildebrandt et al. (18) and Xiao et al. (17) previously reported lowered cDPP enzyme activity in patients with CD and UC, suggesting potential biomarker application of DPP4 for IBD. Moran et al. (40) also reported lower cDPP4 protein expression in patients with CD compared with healthy controls. Most recently, Pinto-Lopes et al. (49) reported decreased DPP4 protein in serum and feces of patients with CD and UC with active disease. The authors proposed that cDPP4 protein could be used as a biomarker for IBD and is a prognosticator of treatment escalation (49). Elevated fecal DPP4 protein has also been reported in patients with CD with ileal active disease, while contrastingly lower fecal DPP4 was reported in patients with UC with active disease (50). In our study, AUROC curve analysis suggested that total cDPP enzyme activity could differentiate active and inactive disease in CD with good sensitivity and specificity. Larger patient cohorts and independent validation are needed to confirm this observation, and whether combination with other serological markers could improve sensitivity and specificity.

DPP4 tissue expression and activity have been investigated in small intestinal disease, such as Crohn's disease and celiac disease (40,51,52); however, tissue expression and activity profiles of all DPPs during IBD have not been characterized previously. In murine experimental colitis, increased expression of DPP8 (13) as well as DPP4 and FAP (53) occurs within affected tissues. Higher levels of DPP4, DPP8, and FAP mRNA, as well as elevated DPP8/9 enzyme activity, were observed in noninflamed colorectal mucosa of patients with IBD compared with non-IBD patients. In small intestinal diseases, reduction in tDPP4 expression and tDPP activity have been associated with the degree of mucosal damage (40,51,52). In the colonic mucosa, changes in DPP expression and activity could be tissue specific or could also be attributed to inflammatory infiltrate. Determining the cell specific expression and activity of DPPs will provide insights into potential targets for therapeutic intervention.

DPP8 and DPP9 have been implicated in immune cell regulation and the inflammatory response. De Vasconcelos et al. (54) reported that DPP8/9 inhibitors can sensitize macrophages to rapid pyroptosis induction and increased secretion of IL-1β and IL-18. Recently, DPP9 has been implicated in suppressing inflammasome formation through interactions between DPP9 and the inflammasome sensors, caspase activation, and recruitment domain-8 (55), nucleotide-binding domain, and leucine-rich repeat pyrin-domain containing protein 1 (56,57). We observed dysregulated DPP8 and DPP9 gene expression in the colonic tissue of patients with IBD and non-IBD patients. Furthermore, the gene expression of these 2 enzymes was found to be strongly correlated, suggesting coregulation.

There were a few study limitations that should be considered when interpreting these findings. The retrospective nature of study 1 means a definitive conclusion about the clinical utility of cDPP enzyme activity as an IBD biomarker cannot be drawn. Prospective studies in larger patient cohorts and independent validation are required to confirm these promising findings. Larger studies would also permit detailed subgroup analysis to determine the optimum clinical utility of dipeptidyl peptidase enzyme activity as a biomarker in IBD. Furthermore, the influence of medication use on DPP activity could not be accurately differentiated in this study. The influence of medications, particularly anti-inflammatory drugs, immunosuppressants, and biologicals, such as anti-tumor necrosis factor agents, should be determined in future work. Finally, fecal calprotectin has emerged as a minimally invasive biomarker for disease activity in IBD (58). Although fecal calprotectin was not routinely collected in the current patient cohort, additional studies could investigate the relationship of cDPP enzyme activity to fecal calprotectin levels.

In conclusion, by discriminating between total cDPP, cDPP4, and residual cDPP activities, we have identified distinct activity profiles between CD and UC with potential biomarker applications. ROC curve analysis suggested good sensitivity and specificity of total and residual cDPP enzyme activity to discriminate patients with IBD with active disease from those in remission. These data encourage future cross-validation studies to develop this observation. The expression and activity profiles of DPP8 and DPP9 in IBD tissues suggest possible immunoregulatory roles for these enzymes. Determining the cell specific expression profile and function of the DPP4 enzyme family during human IBD may identify new opportunities for therapeutic intervention and novel biomarkers to differentiate pathophysiological aspects of the disease.

## CONFLICTS OF INTEREST

**Guarantor of the article:** Roger Yazbeck, PhD.

**Specific author contributions:** S.E.J.: project conceptualization, conducted all experimental work, data analysis, and interpretation, and drafted manuscript; C.A.A.: project conceptualization, data analysis, and editing of manuscript; M.D.G.: contributed to experimental work, data interpretation, and editing of manuscript; P.B.: patient recruitment, access to biological samples, consulted on clinical information, and editing of manuscript; R.N.B.: project conceptualization and editing of manuscript; R.Y.: project conceptualization, data analysis, drafting, and editing of manuscript.

**Financial support:** R.Y. has received funding support from a National Health & Medical Research Council early career fellowship and Flinders University Catherine Marie Enright Kelly fellowship to support the work described in this publication. S.E.J. was supported by a Flinders University Research Scholarship.

**Potential competing interests:** None to report.

**Study Highlights**

**WHAT IS KNOWN**

✓ Inflammatory bowel disease (IBD) is a chronic and debilitating condition.
✓ Objective biomarkers may assist in improving disease management.
✓ The dipeptidyl peptidase (DPP) protease family has been suggested as a possible IBD biomarker.

**WHAT IS NEW HERE**

✓ Circulating DPP enzyme activity differentiated active disease from remission in patients with IBD.
✓ Low circulating DPP enzyme activity is associated with elevated plasma C-reactive protein.
✓ DPP enzyme activity could have utility as serological markers of inflammation in IBD.

## Supplementary Material

SUPPLEMENTARY MATERIAL
