# Supplementary material for: Circulating Dipeptidyl Peptidase Activity Is a Potential Biomarker for Inflammatory Bowel Disease
Source: Clin Transl Gastroenterol. 2022 Jan 19;13(1):e00452. doi: 10.14309/ctg.0000000000000452 (PMC8806366; doi:10.14309/ctg.0000000000000452)
Supplement: SUPPLEMENTARY MATERIAL [file ct9-13-e00452-s001.docx]

**Supplementary Table** **1.** Primer sequences used to synthesize external gene standards and to quantify DPP mRNA expression within human gastrointestinal biopsies.

|  | Forward Primer (5’–3’) | Reverse Primer (5’–3’) | Product (bp) |
| --- | --- | --- | --- |
| DPP4 | ACGCCGACGATGAAGACACCG | TTCAGCAGACCACGGGCACG | 95 |
| FAP | ATGGGGCTGGTCCTATGGAGGAT | GCTGGAGACTGGAGCCACTGC | 97 |
| DPP8 | ACATGATGGCTAAGGCACCAATGA | CTGTTCTCACCAGACATGGCAAGG | 106 |
| DPP9 | TTGCTCTGACCAGCGGTGAATG | GCCTCATAGCTGACCACGTAGA | 142 |
| TATA | CGAAACGCCGAATATAATCCCAAGCG | GCCAGTCTGGATGTTCTTCA CTC TT | 137 |
| PPIA | GGCAAATGCTGGACCCAACACA | TGCTGGTCTTGCCATTCCTGGA | 161 |
