# Supplementary figures and images for: Circulating Dipeptidyl Peptidase Activity Is a Potential Biomarker for Inflammatory Bowel Disease
Source: Clin Transl Gastroenterol. 2022 Jan 19;13(1):e00452. doi: 10.14309/ctg.0000000000000452 (PMC8806366; doi:10.14309/ctg.0000000000000452)

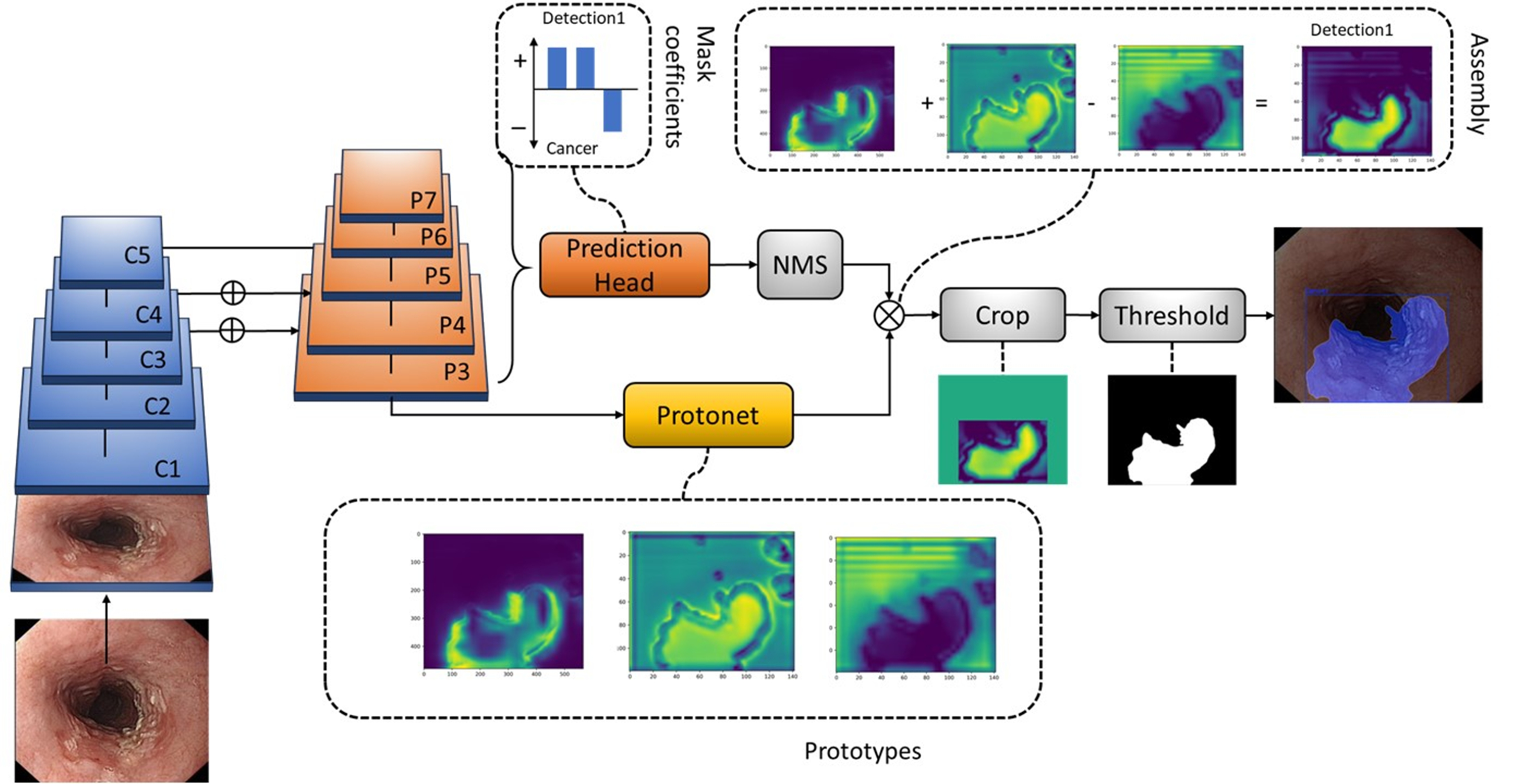

Supplement: SUPPLEMENTARY MATERIAL [file ct9-13-e00452-s002.tif]

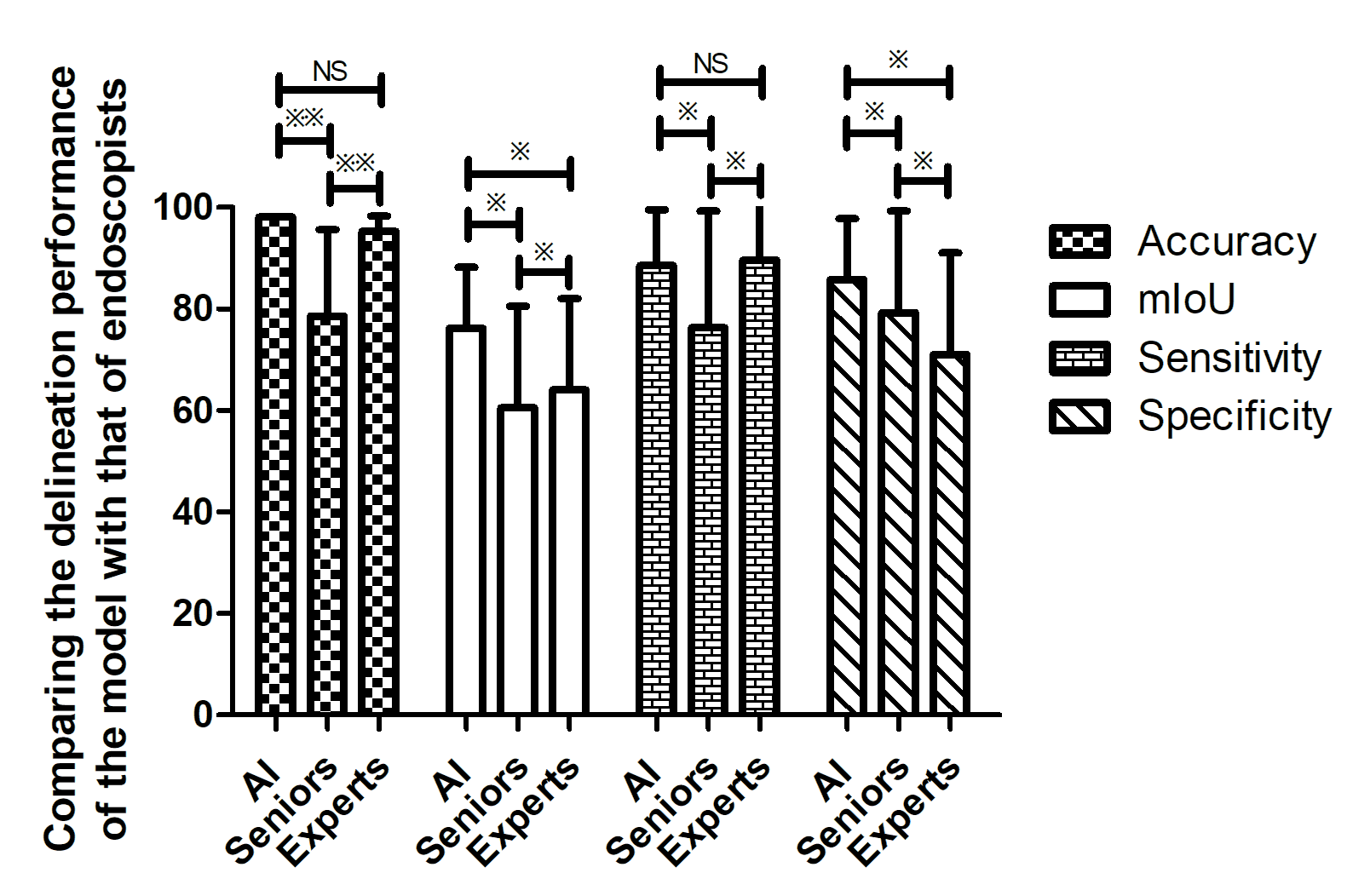

Supplement: SUPPLEMENTARY MATERIAL [file ct9-13-e00452-s004.tif]
