# Supplementary material for: Circulating Dipeptidyl Peptidase Activity Is a Potential Biomarker for Inflammatory Bowel Disease
Source: Clin Transl Gastroenterol. 2022 Jan 19;13(1):e00452. doi: 10.14309/ctg.0000000000000452 (PMC8806366; doi:10.14309/ctg.0000000000000452)
Supplement: SUPPLEMENTARY MATERIAL [file ct9-13-e00452-s003.docx]

**Supplemental Table.** Other pancreatic disorders

| **VARIABLE** | **CONTROL** | **CASE** |
| --- | --- | --- |
| **ANY DIABETES MELLITUS** | 462,233 (92.0) | 40,252 (8.0) |
| **T1 DIABETES MELLITUS** **(INSULIN DEPENDENT)** | 497,693 (99.0) | 4,792 (1.0) |
| **T2 DIABETES MELLITUS (NON-INSULIN DEPENDENT)** | 46,7174 (93.0) | 35,311 (7.0) |
| **PANCREATIC CANCER:** | 500,656 (99.6) | 1,829 (0.4) |
| **CYSTIC FIBROSIS** | 502,346 (99.98) | 129 (0.02) |
